# Diagnostic and prognostic value of autophagy-related key genes in sepsis and potential correlation with immune cell signatures

**DOI:** 10.3389/fcell.2023.1218379

**Published:** 2023-08-28

**Authors:** Li Yang, Lin Zhou, Fangyi Li, Xiaotong Chen, Ting Li, Zijun Zou, Yaowei Zhi, Zhijie He

**Affiliations:** ^1^ Department of Critical Care Medicine, Sun Yat-Sen Memorial Hospital, Sun Yat-Sen University, Guangzhou, Guangdong, China; ^2^ Department of Health Management Center, Sun Yat-Sen Memorial Hospital, Sun Yat-Sen University, Guangzhou, Guangdong, China

**Keywords:** sepsis, autophagy, immune cell signatures, bioinformatics analysis, diagnostic, prognostic

## Abstract

**Background:** Autophagy is involved in the pathophysiological process of sepsis. This study was designed to identify autophagy-related key genes in sepsis, analyze their correlation with immune cell signatures, and search for new diagnostic and prognostic biomarkers.

**Methods:** Whole blood RNA datasets GSE65682, GSE134347, and GSE134358 were downloaded and processed. Differential expression analysis and weighted gene co-expression network analysis (WGCNA) were used to identify autophagy-related key genes in sepsis. Then, key genes were analyzed by functional enrichment, protein-protein interaction (PPI), transcription factor (TF)-gene and competing endogenous RNA (ceRNA) network analysis. Subsequently, key genes with diagnostic efficiency and prognostic value were identified by receiver operating characteristic (ROC) curves and survival analysis respectively. The signatures of immune cells were estimated using CIBERSORT algorithm. The correlation between significantly different immune cell signatures and key genes was assessed by correlation analysis. Finally, key genes with both diagnostic and prognostic value were verified by RT-qPCR.

**Results:** 14 autophagy-related key genes were identified and their TF-gene and ceRNA regulatory networks were constructed. Among the key genes, 11 genes (ATIC, BCL2, EEF2, EIF2AK3, HSPA8, IKBKB, NLRC4, PARP1, PRKCQ, SH3GLB1, and WIPI1) had diagnostic efficiency (AUC > 0.90) and 5 genes (CAPN2, IKBKB, PRKCQ, SH3GLB1 and WIPI1) were associated with survival prognosis (*p*-value < 0.05). IKBKB, PRKCQ, SH3GLB1 and WIPI1 had both diagnostic and prognostic value, and their expression were verified by RT-qPCR. Analysis of immune cell signatures showed that the abundance of neutrophil, monocyte, M0 macrophage, gamma delta T cell, activated mast cell and M1 macrophage subtypes increased in the sepsis group, while the abundance of resting NK cell, resting memory CD4^+^ T cell, CD8^+^ T cell, naive B cell and resting dendritic cell subtypes decreased. Most of the key genes correlated with the predicted frequencies of CD8^+^ T cells, resting memory CD4^+^ T cells, M1 macrophages and naive B cells.

**Conclusion:** We identified autophagy-related key genes with diagnostic and prognostic value in sepsis and discovered associations between key genes and immune cell signatures. This work may provide new directions for the discovery of promising biomarkers for sepsis.

## Introduction

Sepsis refers to the life-threatening organ dysfunction caused by a dysregulated host response to infection (Singer et al., 2016). According to conservative estimates, there were 48.9 million patients with sepsis and 11.0 million sepsis related deaths worldwide in 2017, representing 19.7% of all global deaths (Rudd et al., 2020). Due to its high morbidity and mortality, sepsis has become an important health problem. But sepsis is treatable, and early intervention can improve treatment outcomes and reduce mortality (Liu et al., 2017; Rhodes et al., 2017; Seymour et al., 2017; Evans et al., 2018). Therefore, early diagnosis and prognostic prediction of sepsis are particularly important.

Autophagy is an evolutionarily conserved biological process responsible for degrading unwanted cytoplasmic components and invading microorganisms. It plays a central role in cytoplasmic quality control, cellular metabolism, innate and adaptive immunity (Deretic, 2021). In sepsis, autophagy has protective effect on heart, kidney, lung and brain, and has damaging effect on skeletal muscles (Stana et al., 2017; Sun et al., 2018; Zhuang et al., 2020; Wang et al., 2021b; Chen et al., 2021). As a complex immune response that changes over time, the core mechanism of sepsis is dysregulated innate and adaptive immune responses of the host. The functional status and distribution of immune cells determine the initiation, development and prognosis of sepsis. Autophagy plays a protective role in immune cells by regulating various cellular receptors and signaling pathways in order to maintain immune homeostasis in sepsis. Enhanced autophagic activity of neutrophils increases neutrophil extracellular trap (NET) formation, which is important for maintaining appropriate neutrophil function during sepsis (Park et al., 2017). Treatment with lipopolysaccharide (LPS) in autophagy-deficient macrophages resulted in increased secretion of macrophage mobility inhibitory factor (MIF) and aggravated inflammation (Lee et al., 2016). Suppressing the autophagic activity of dendritic cells can negatively regulate their immune function (Liu et al., 2021). T-cell-specific mTOR deletion improves cell survival in mice with fatal fungal sepsis by enhancing autophagy (Wang et al., 2019; Wang et al., 2021a).

Given the important role of autophagy in sepsis, we speculate that the exploration of autophagy-related genes (ARGs) will provide potential diagnostic and prognostic biomarkers for the treatment of sepsis. Stunning advances in sequencing technology and bioinformatics have provided great convenience for the study of sepsis at the genetic level. Whole blood RNA, which is mainly derived from blood immune cells, is our first choice due to its easy accessibility and ability to represent the immune response in sepsis. Therefore, we conducted a comprehensive bioinformatics analysis to explore the role of peripheral blood ARGs in sepsis.

In this study, through comprehensive analysis, autophagy-related key genes in sepsis were identified, and the key genes with diagnostic efficiency and prognostic value were obtained. Furthermore, the signatures of immune cells and their correlation with key genes were clarified. The study may provide new ideas for further exploring the pathophysiological mechanism, diagnosis and treatment of sepsis.

## Materials and methods

### Data collection and processing

All microarray datasets (GSE65682, GSE134347 and GSE134358) (Scicluna et al., 2015; Scicluna et al., 2018; Scicluna et al., 2020) were obtained from the GEO database (https://www.ncbi.nlm.nih.gov/geo/). GSE65682 (685 sepsis patients and 42 healthy controls) that mainly contained whole blood mRNA data was generated by means of Affymetrix Human Genome U219 Array (GPL13667). GSE134347 (156 sepsis patients and 83 healthy controls) that mainly contained whole blood mRNA and lncRNA data was examined by Affymetrix Human Transcriptome Array 2.0 (GPL17586). The GSE134358 (158 sepsis patients and 82 healthy controls) was a whole blood miRNA expression profile dataset detected by Affymetrix Multispecies miRNA-4 Array (GPL21572). Raw data was read into the R software (version 4.2.0) using the affy or oligo package (Gautier et al., 2004; Carvalho and Irizarry, 2010). Robust Multi-array Average (RMA) (Irizarry et al., 2003) was used for background correction and normalization of the array data. The resultant probe intensities were filtered using the genefilter package by a 0.5 variance cutoff. The ComBat method of sva package (Leek et al., 2012) was used to evaluated and corrected the batch effect. Finally, annotation files downloaded from Affymetrix official website were used for probe annotation.

### Autophagy-related genes (ARGs) and differential expression analysis

From The Human Autophagy Database (http://www.autophagy.lu/index.html), we obtained the list of ARGs containing 222 genes. According to this gene list, the ARGs in GSE65682 and GSE134347 were extracted.

Differential expression analysis was performed by limma package (Ritchie et al., 2015). Adjusted *p*-value < 0.05 and |log_2_ fold change (FC)| > 0.5 was set as the screening threshold. Volcano plots of the differentially expressed mRNAs, lncRNAs, miRNAs and ARGs (DEmRNAs, DElncRNAs, DEmiRNAs and DEARGs) were visualized by ggplot2 package. DEmRNAs and DEARGs shared by GSE65682 and GSE134347 were visualized using the VennDiagram package (Chen and Boutros, 2011).

### Weighted gene co-expression network analysis (WGCNA) and identification of autophagy-related key genes in sepsis

All expressed mRNAs in GSE65682 and GSE134347 were extracted and analyzed by WGCNA package (Langfelder and Horvath, 2008). Sample cluster analysis was performed to identify and remove outlier samples. The appropriate soft threshold (β) was selected to construct the network by one-step method, and the cluster dendrogram was drawn to visualize the modules represented by different colors. Subsequently, the correlation between groups and modules was calculated by Spearman correlation analysis, and the genes of the three modules with the highest absolute values of correlation coefficients were selected as sepsis-related genes (SRGs). Finally, the shared SRGs and the shared DEARGs of GSE65682 and GSE134347 were intersected to obtain the autophagy-related key genes.

### Functional enrichment analysis and protein-protein interaction (PPI) analysis

The clusterProfiler package (Yu et al., 2012) was used to conduct Gene Ontology (GO) (Harris et al., 2004) and Kyoto Encyclopedia of Genes and Genomes (KEGG) (Kanehisa et al., 2022) pathway enrichment analysis of key genes. The enrichment results were visualized by ggplot2.

PPI network was created using the online STRING database (http://www.string-db.org/, version 11.5) (Szklarczyk et al., 2021) with the interaction scores > 0.4, and then analyzed and visualized by Cytoscape (version 3.9.1) (Shannon et al., 2003). The Cytoscape plug-in CytoNCA (Tang et al., 2015) was employed to rank nodes based on betweenness.

### Construction of transcription factor (TF)-gene regulatory network and competing endogenous RNA (ceRNA) regulatory network

The autophagy-related key genes were uploaded to the online platform NetworkAnalyst (https://www.networkanalyst.ca/, version 3.0) (Zhou et al., 2019) and analysed using the TF-gene interactions modules based on the ENCODE database (https://www.encodeproject.org/) (Luo et al., 2020) to identify the upstream TFs. Then the network was visualized by Cytoscape.

The ceRNA theory suggests that lncRNAs can regulate mRNAs by binding and neutralizing the matched miRNAs. Following this theory, we constructed the ceRNA regulatory network. First, we used miRDB (https://www.mirdb.org/) (Chen and Wang, 2020), miRTarBase (https://mirtarbase.cuhk.edu.cn/∼miRTarBase/miRTarBase_2019/php/index.php) (Huang et al., 2020) and TargetScan (https://www.targetscan.org/) (Agarwal et al., 2015) databases to obtain the DEmiRNAs targeting autophagy-related key genes. Next, the miRNAs matching the DElncRNAs were obtained using the miRCode database (http://www.mircode.org/) (Jeggari et al., 2012). Finally, the ceRNA regulatory network of autophagy-related key genes was obtained by intersecting the above two results and was visualized by the ggalluvial package.

### Receiver operating characteristic (ROC) curve analysis

ROC curve analysis was performed using pROC package (Robin et al., 2011). The diagnostic value of autophagy-related key genes in sepsis was evaluated by the area under the curve (AUC). Genes with AUC > 0.90 were considered to have diagnostic value in sepsis.

### Survival analysis

We extracted samples with survival information from GSE65682 for survival analysis. All samples were grouped based on the median expression of each key gene. Survival and survminer packages were used to assess the influence of key genes on 28-day survival and draw Kaplan-Meier (K-M) survival curves. The genes associated with 28-day survival (*p*-value < 0.05) were considered to have prognostic value in sepsis.

### Assessment of immune cell signatures

The array of immune cells was estimated by the CIBERSORT algorithm based on the leukocyte signature matrix (LM22) at 100 permutations (Newman et al., 2015). Next, we compared the expression of each immune cell subtype RNA signature between the sepsis group and the healthy control group to determine which cell subtypes may be significantly different in their abundance. Finally, the correlation between autophagy-related key genes and significantly different immune cell signatures was calculated by Spearman correlation analysis using the psych package, and the results were visualized by ggplot2.

### Real-time quantitative polymerase chain reaction (RT-qPCR)

11 healthy controls and 11 sepsis patients, all older than 18 years, were enrolled in this study. All patients met the diagnostic criteria of sepsis 3.0 and were admitted to the intensive care unit of Sun Yat-Sen Memorial Hospital of Sun Yat-Sen University from September 2022 to October 2022. The clinical characteristics of the individuals participating in the validation experiment are described in Supplementary Table S1.

Peripheral blood samples of enrolled patients were collected within 24 h after admission. Following the manufacturer’s instructions, RNA was extracted from whole blood using RNAprep Pure Hi-Blood Kit (DP443, TIANGEN Biotech, Beijing, China). Then, reverse transcription reaction and RT-qPCR were performed using PrimeScript™ RT reagent Kit with gDNA Eraser (RR047, TaKaRa, Dalian, China) and TB Green Premix Ex Taq™ (RR820, TaKaRa, Dalian, China), respectively. ACTB was used as the reference gene and the relative expression was calculated by 2^−ΔΔCT^ Method. Primer sequences are listed in Supplementary Table S2.

### Statistical analysis

Comparison of continuous variables between two groups was performed in SPSS software (version 24.0). Student’s t-test and Mann-Whitney *U*-test were applied to normal and non-normal distribution data, respectively. The results of the RT-qPCR were plotted in GraphPad Prism (version 8.0). Other data in this article were calculated automatically by R software (version 4.2.0) or online database mentioned above. *p*-value < 0.05 was considered statistically significant.

## Results

### Differentially expressed mRNAs, lncRNAs, miRNAs and ARGs in sepsis

**FIGURE 1:**
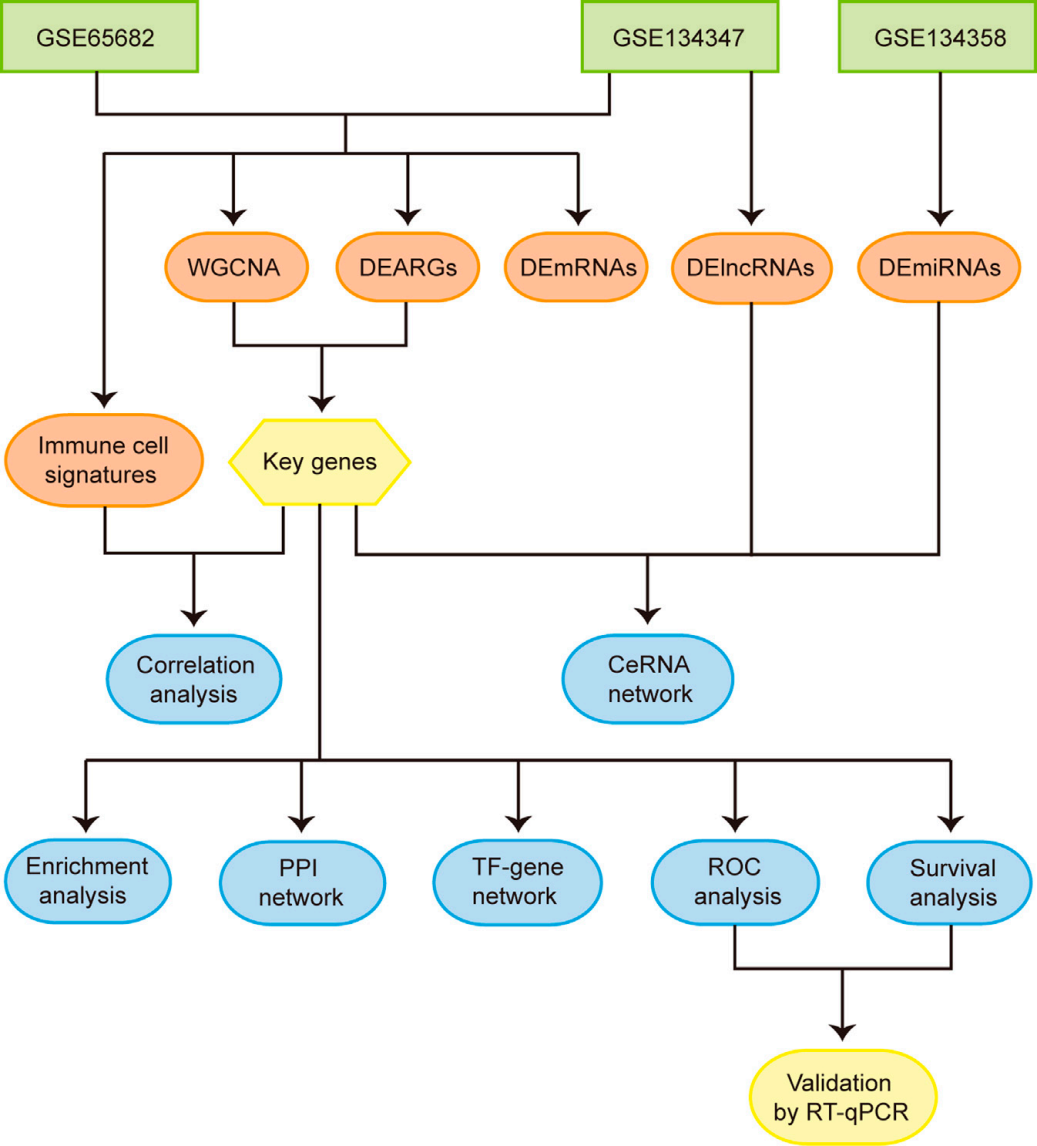
Flowchart of the overall study. Abbreviations: WGCNA, weighted gene co-expression network analysis; DEARGs, differentially expressed autophagy-related genes; DEmRNAs, differentially expressed mRNAs; DElncRNAs, differentially expressed lncRNAs; DEmiRNAs, differentially expressed miRNAs; CeRNA, competing endogenous RNA; PPI, protein-protein interaction; TF, transcription factor; ROC, receiver operating characteristic.

**FIGURE 2:**
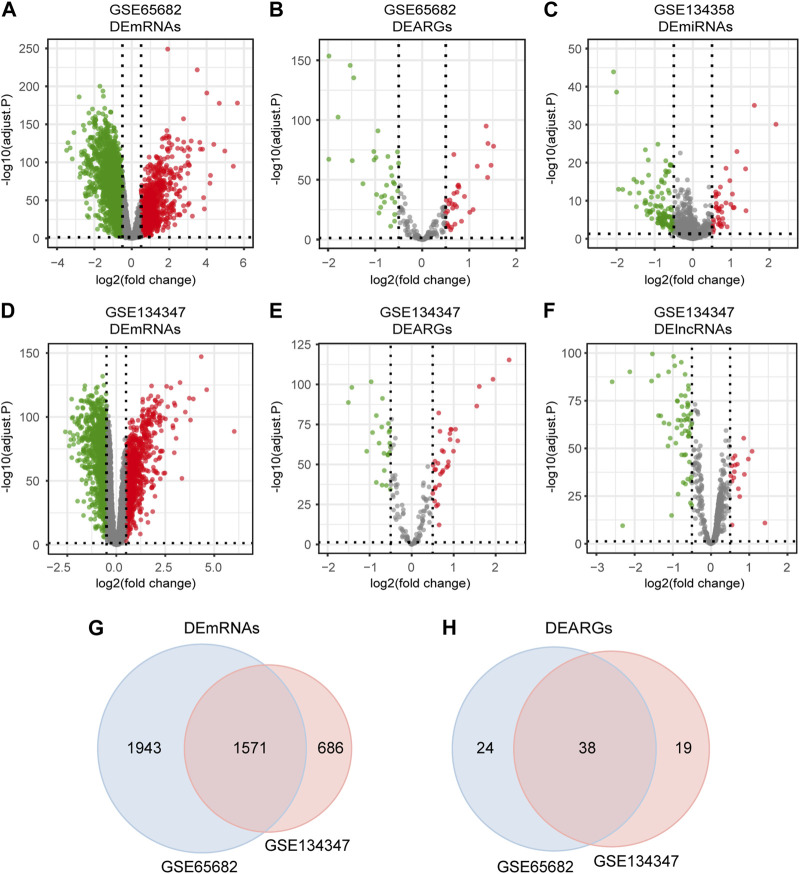
Differentially expressed mRNAs, lncRNAs, miRNAs and ARGs in sepsis. **(A,B)** Volcano plots of DEmRNAs and DEARGs in GSE65682. **(C)** Volcano plot of DEmiRNAs in GSE134358. **(D–F)** Volcano plots of DEmRNAs, DEARGs and DElncRNAs in GSE134347. **(G)** Venn diagram of overlapping DEmRNAs between GSE65682 and GSE134347. **(H)** Venn diagram of overlapping DEARGs between GSE65682 and GSE134347.

The detailed workflow of the overall study is shown in Figure 1. After data processing, we obtained 11,476 genes in GSE65682, 12,985 genes in GSE134347 and 1,695 genes in GSE134358. By referring to comprehensive gene annotation and long non-coding RNA gene annotation (V41) downloaded from GENCODE (https://www.gencodegenes.org/human/), we obtained 10,626 mRNAs and 75 lncRNAs in GSE65682, 9044 mRNAs and 647 lncRNAs in GSE134347. Due to the small number of lncRNAs contained in GSE65682, the lncRNA data of GSE134347 was used for differential expression analysis. By intersecting mRNAs with the list of ARGs, we extracted 188 ARGs in GSE65682 and 153 ARGs in GSE134347. Subsequently, through differential expression analysis between sepsis group and healthy control group, we obtained 3,514 DEmRNAs and 62 DEARGs in GSE65682, 2,257 DEmRNAs, 76 DElncRNAs and 57 DEARGs in GSE134347, and 150 DEmiRNAs in GSE134358 (Figures 2A–F). In addition, there were 1,571 DEmRNAs and 38 DEARGs shared by GSE65682 and GSE134347 (Figures 2G, H).

### WGCNA and screening for autophagy-related key genes in sepsis

**FIGURE 3:**
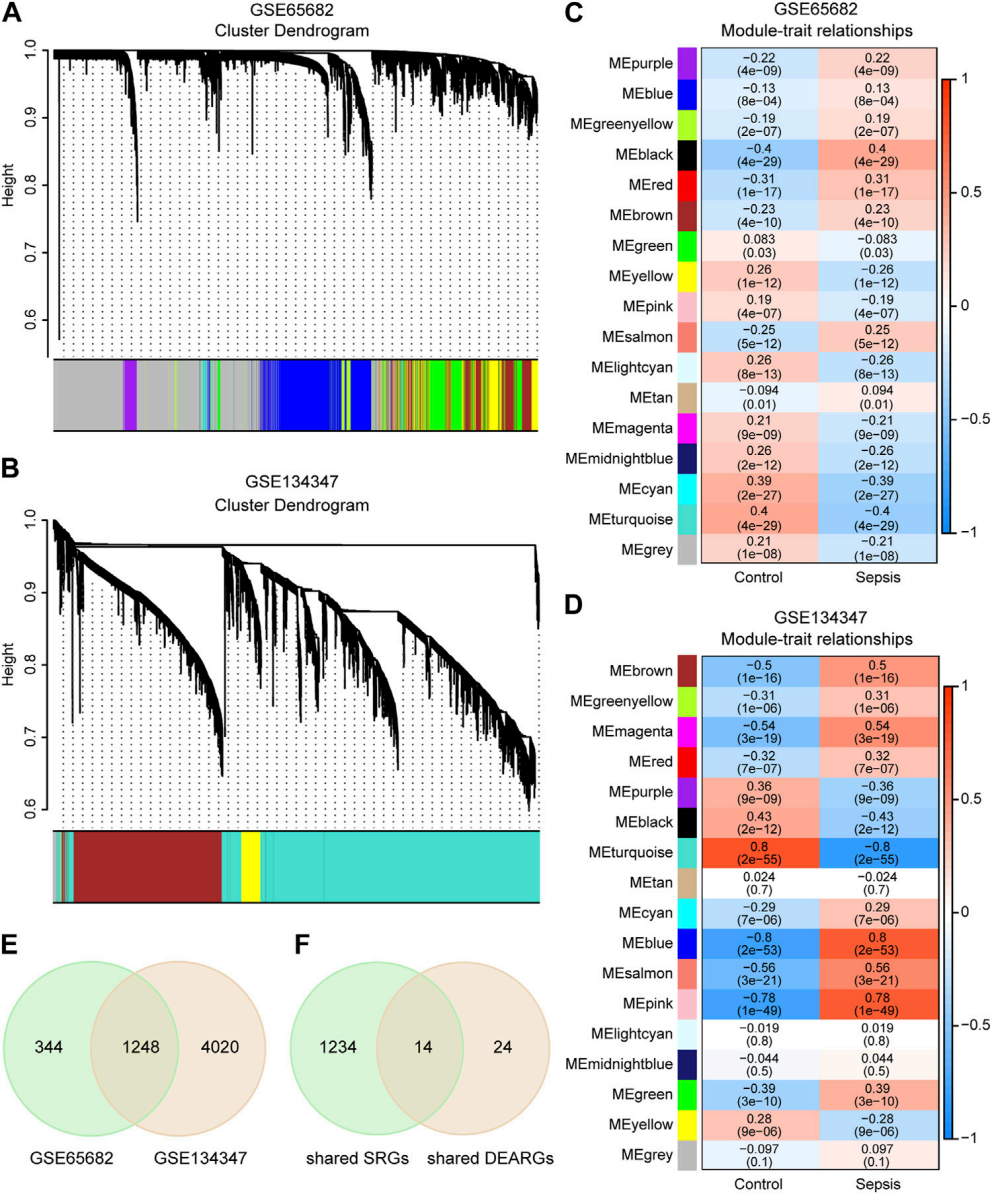
Identification of autophagy-related key genes in sepsis. **(A,B)** Clustering dendrograms and assigned module colors of mRNAs in GSE65682 and GSE134347. **(C,D)** Module-trait relationships of GSE65682 and GSE134347. The correlation coefficient and *p*-value are displayed in the grid of the heatmap. **(E)** Venn diagram of overlapping sepsis-related genes (SRGs) between GSE65682 and GSE134347. **(F)** Identification of autophagy-related key genes by overlapping shared SRGs and shared DEARGs through Venn diagram.

By sample cluster analysis, 10 outlier samples in GSE65682 were removed, and no obvious outlier samples were found in GSE134347. The power of β = 12 in GSE65682 (R^2^ = 0.89, slope = −2.26) and β = 8 in GSE134347 (R^2^ = 0.86, slope = −1.28) were selected. Then, the network was constructed and 16 gene modules with different colors were obtained in each dataset (Figures 3A, B). Black, turquoise and cyan were the three modules with the highest absolute values of correlation coefficients in GSE65682, containing 1,592 SRGs (Figure 3C). Similarly, turquoise, blue and pink were the three modules with the highest absolute values of correlation coefficients in GSE134347, containing 5,268 SRGs (Figure 3D). There were 1,248 SRGs shared by GSE65682 and GSE134347 (Figure 3E). By intersecting shared SRGs and shared DEARGs, we identified 14 autophagy-related key genes in sepsis (Figure 3F).

### Functional enrichment and PPI network analysis of autophagy-related key genes

**FIGURE 4:**
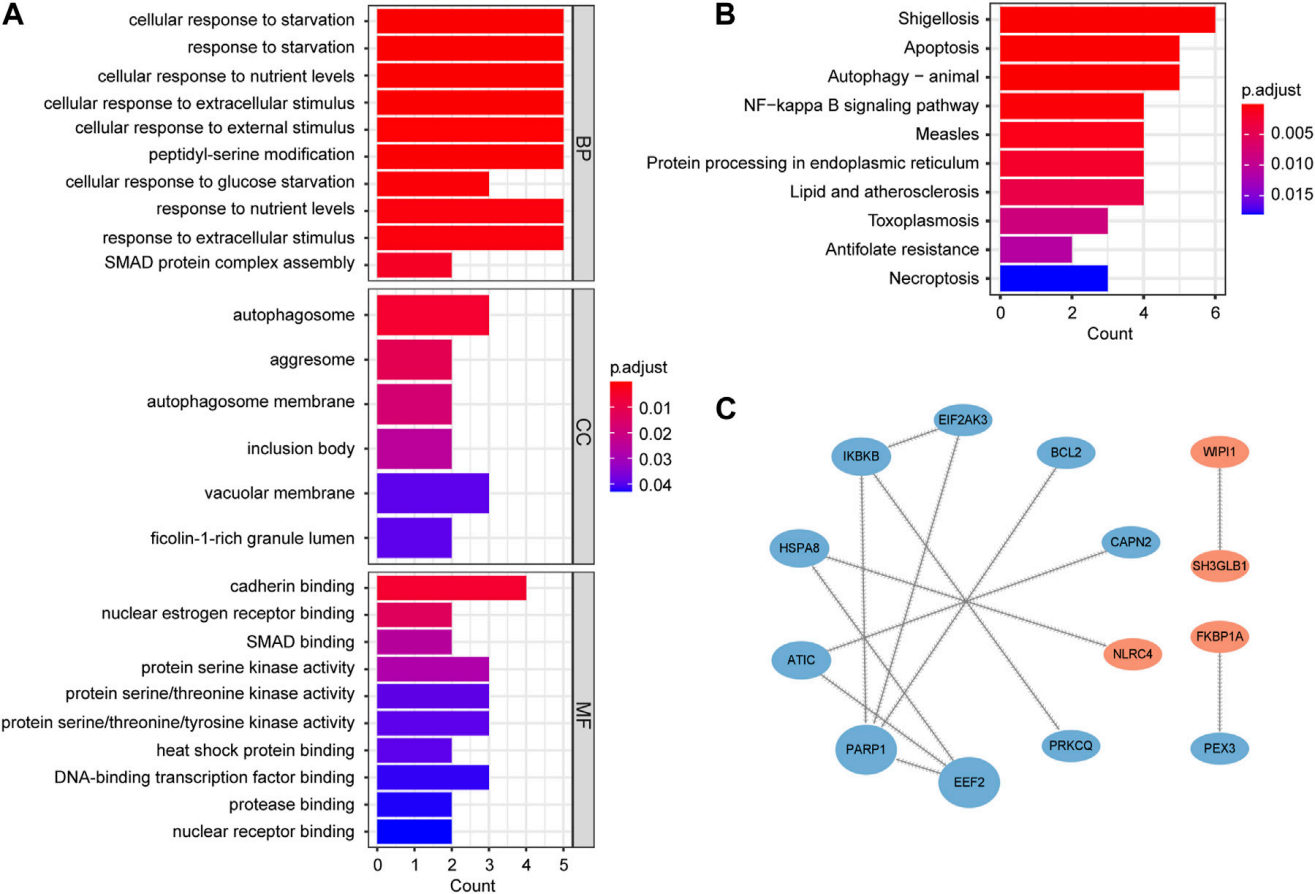
Functional enrichment and PPI network analysis of autophagy-related key genes. **(A)** Gene Ontology (GO) enrichment analysis results, including biological process (BP), cellular component (CC), and molecular function (MF). **(B)** Kyoto Encyclopedia of Genes and Genomes (KEGG) pathway enrichment analysis results. **(C)** PPI network. The nodes are ranked by betweenness. Blue nodes represent downregulated genes and red nodes represent upregulated genes.

GO enrichment analysis includes biological processes (BP), molecular functions (MF) and cellular components (CC). BP analysis indicated that key genes were mainly involved in regulation of cellular response to starvation, response to starvation, cellular response to nutrient levels and cellular response to extracellular stimulus. CC analysis showed that autophagosome, aggresome and autophagosome membrane were the top three enriched terms. In MF analysis, these genes were primarily enriched in cadherin binding, nuclear estrogen receptor binding and SMAD binding (Figure 4A; Supplementary Table S3). The results of KEGG analysis primarily converged on the shigellosis, apoptosis, autophagy-animal and NF-kappa B signaling pathway (Figure 4B; Supplementary Table S4).

In order to study the interactions of key genes, we constructed a PPI network containing 14 nodes and 12 edges. The size and order of nodes in the network were determined by betweenness (Figure 4C).

### TF-gene regulatory network and ceRNA regulatory network of autophagy-related key genes

**FIGURE 5:**
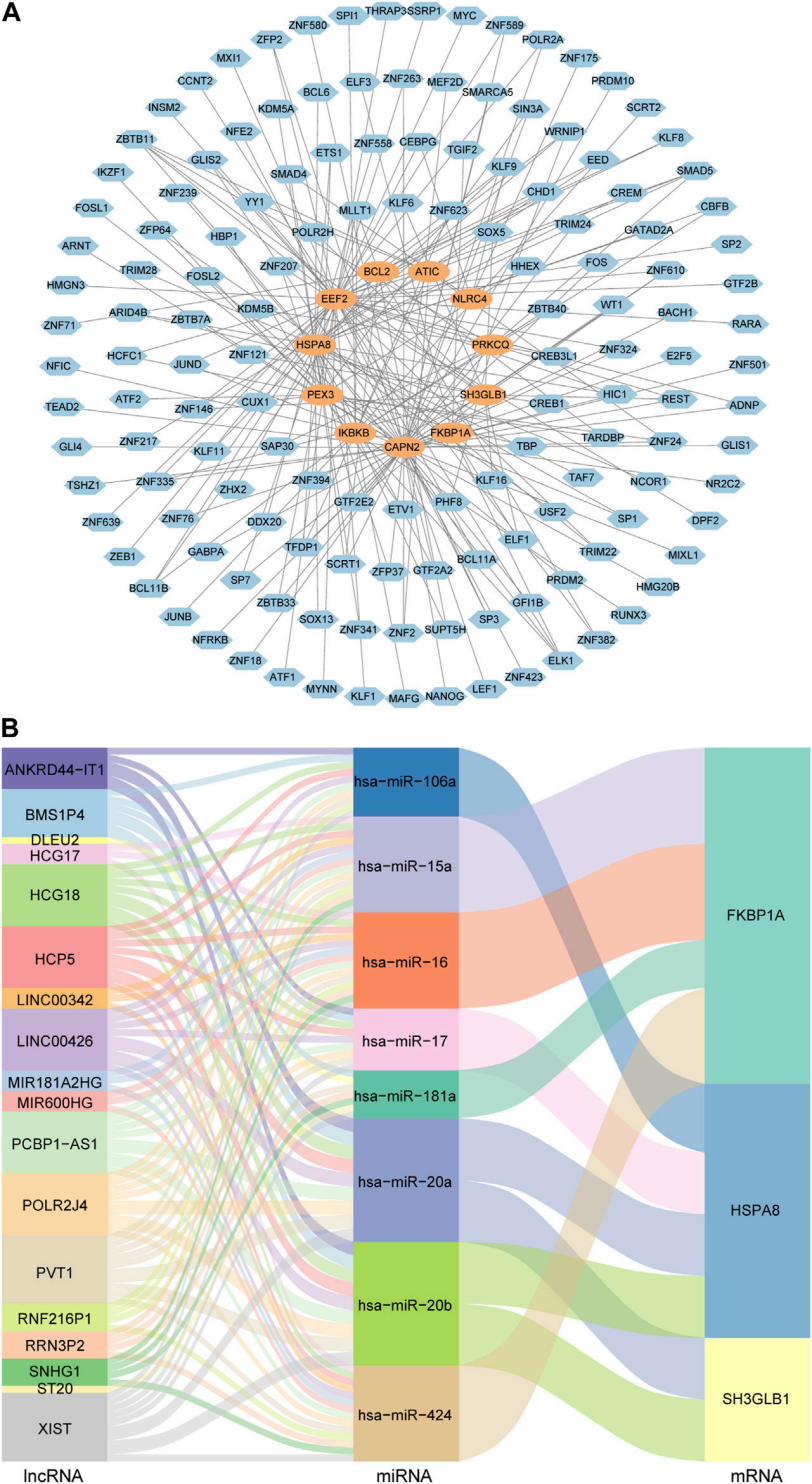
TF-gene regulatory network and ceRNA regulatory network of autophagy-related key genes. **(A)** Diagram of the regulatory network between TFs and key genes. Blue nodes represent TFs and orange nodes represent key genes. **(B)** Sankey diagram of the ceRNA regulatory network. Each rectangle in the diagram represents a gene, and the height of the rectangle indicates the gene’s connection degree.

The TF-gene regulatory network was comprised of 154 nodes and 215 edges. In detail, these nodes were combined by 11 key genes and 143 TFs. Each key gene was regulated by multiple TFs. Among them, CAPN2 was regulated by 56 TFs and EEF2 was regulated by 45 TFs. In addition, 52 TFs regulated more than one key gene, among which ZBTB11 could regulate 5 genes simultaneously (Figure 5A).

In order to understand the regulatory relationships, ceRNA interaction analysis was performed on the obtained 14 key genes, 150 DEmiRNAs and 76 DElncRNAs. And a ceRNA regulatory network consisting of 3 key genes, 8 miRNAs and 18 lncRNAs was constructed. The detailed lncRNA-miRNA-mRNA interaction relationship was visualized by Sankey diagram (Figure 5B).

### Diagnostic value of autophagy-related key genes for sepsis

**FIGURE 6:**
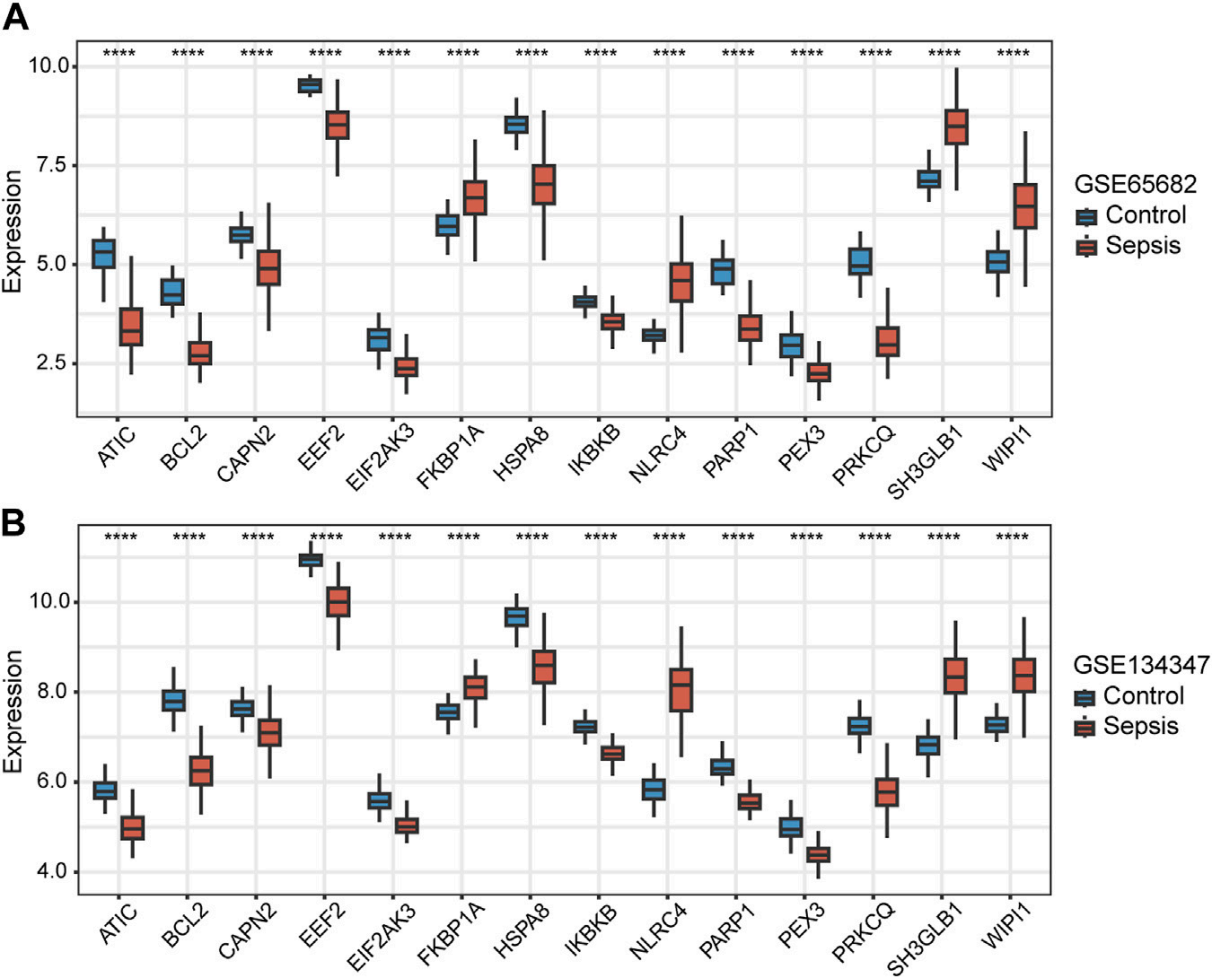
Differences in the expression of autophagy-related key genes between control and sepsis groups in GSE65682 and GSE134347. Student’s t-test or Mann-Whitney *U*-test were used to compare the differences between the two groups. ****p < 0.0001.

**FIGURE 7:**
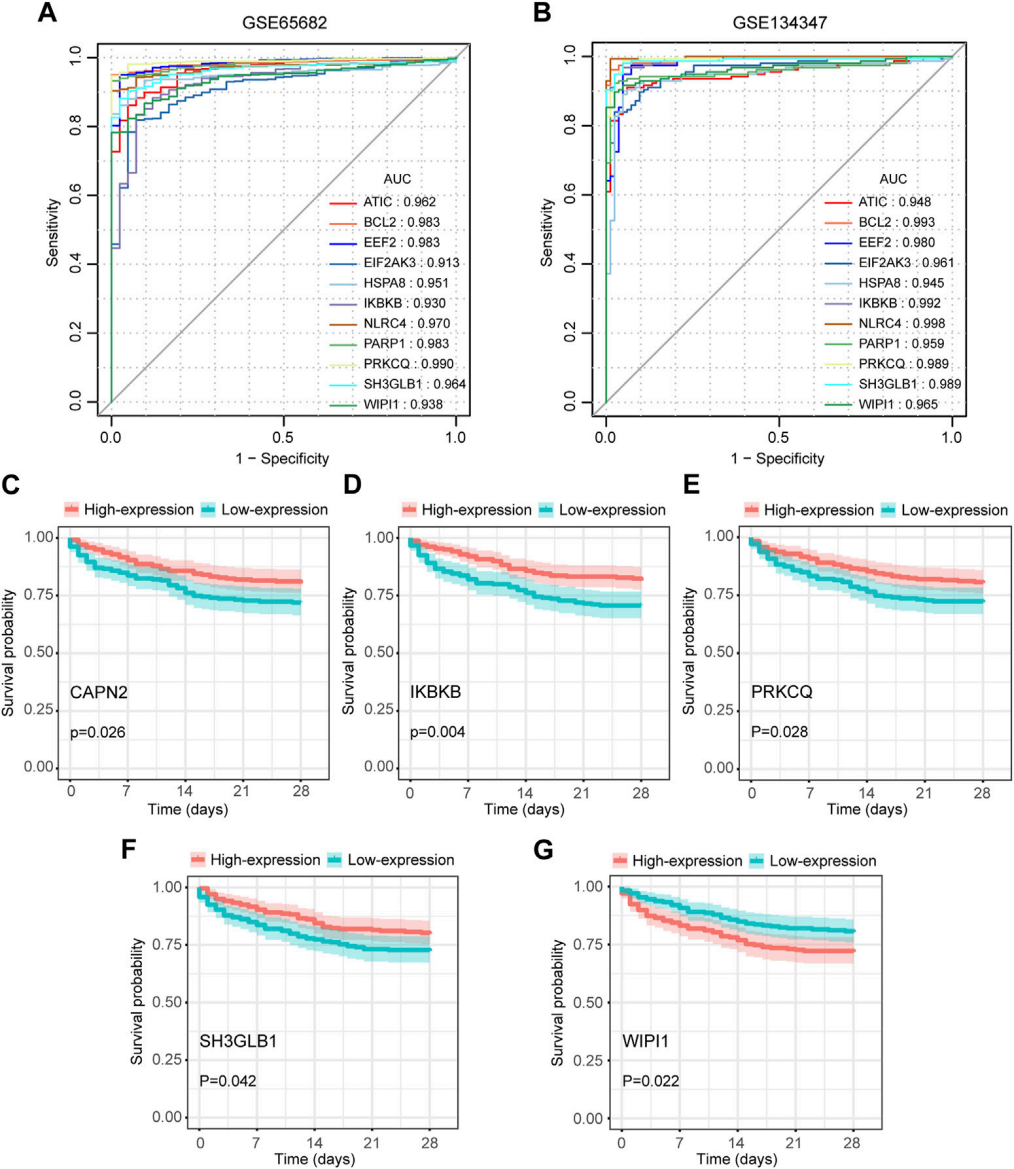
Diagnostic and prognostic capabilities of autophagy-related key genes. **(A,B)** ROC curves of key genes with AUC greater than 0.90 in GSE65682 and GSE134347. **(C–G)** Kaplan-Meier (K-M) curves of key genes with significant differences in survival analysis.

Among the 14 key genes identified, the expressions of FKBP1A, NLRC4, SH3GLB1 and WIPI1 were increased in the sepsis group, while other key genes were decreased (Figures 6A, B). This result suggested that the activation of FKBP1A, NLRC4, SH3GLB1 and WIPI1 and the inactivation of other key genes might indicate the occurrence of sepsis. Further ROC curve analysis showed that a total of 11 key genes (ATIC, BCL2, EEF2, EIF2AK3, HSPA8, IKBKB, NLRC4, PARP1, PRKCQ, SH3GLB1, and WIPI1) had AUCs greater than 0.90 both in GSE65682 and GSE134347, and these genes were considered to have diagnostic efficacy for sepsis (Figures 7A, B).

### Prognostic value of autophagy-related key genes for sepsis

We extracted 477 samples with survival information (363 survivors and 114 nonsurvivors) from GSE65682. Survival analysis suggested that the aberrant expression levels of CAPN2, IKBKB, PRKCQ, SH3GLB1, and WIPI1 were associated with the 28-day survival. Moreover, patients with high expression of CAPN2, IKBKB, PRKCQ, and SH3GLB1 had a higher 28-day survival, while WIPI1 had an opposite effect (Figures 7C–G). Therefore, these 5 key genes were considered to have prognostic value for sepsis.

### Immune cell signatures and their correlation with autophagy-related key genes

**FIGURE 8:**
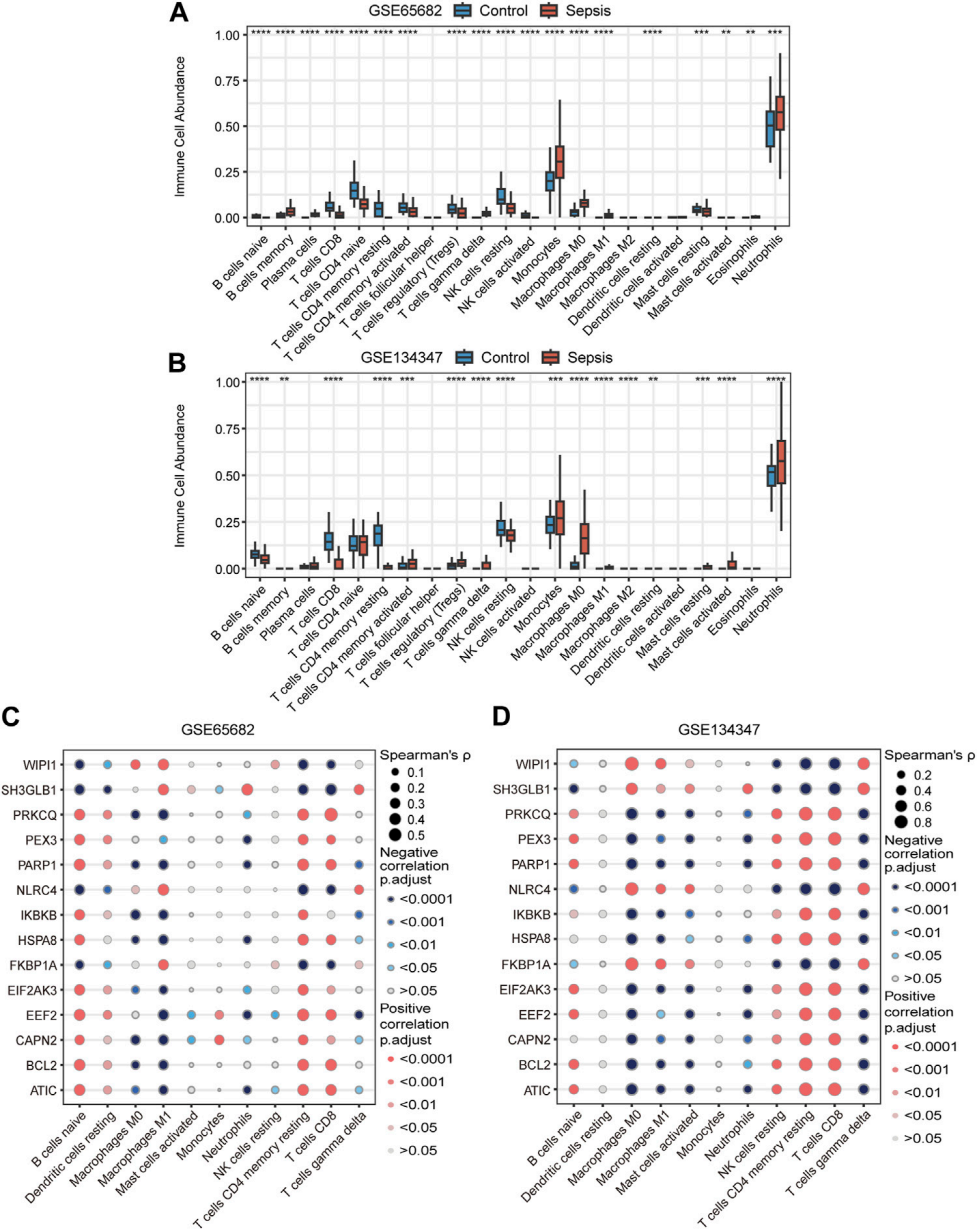
Immune cell signatures and their correlation with autophagy-related key genes. **(A,B)** Differences in immune cell abundance between control and sepsis groups in GSE65682 and GSE134347. Student’s t-test or Mann-Whitney *U*-test were used to compare the two groups. **p < 0.01, ***p < 0.001, ****p < 0.0001. **(C,D)** Correlation heatmaps of key genes and significantly different immune cell signatures in GSE65682 and GSE134347.

Based on RNA signatures the abundance of 19 immune cell subtypes in GSE65682 and 16 subtypes in GSE134347 were significantly different between the sepsis and healthy control groups (Figures 8A, B). The directionality of 11 significantly different subtypes was shared between the two datasets. Neutrophil, monocyte, M0 macrophage, gamma delta (γδ) T cell, activated mast cell and M1 macrophage signatures were more abundant in the sepsis group, while resting NK cell, resting memory CD4^+^ T cell, CD8^+^ T cell, naive B cell and resting dendritic cell signatures were more abundant in the healthy control group. Additionally, Spearman correlation analysis showed that most of the key genes correlated with the predicted frequencies of CD8^+^ T cells, resting memory CD4^+^ T cells, M1 macrophages and naive B cells (Figures 8C, D).

### Validation of selected key genes by RT-qPCR

**FIGURE 9:**
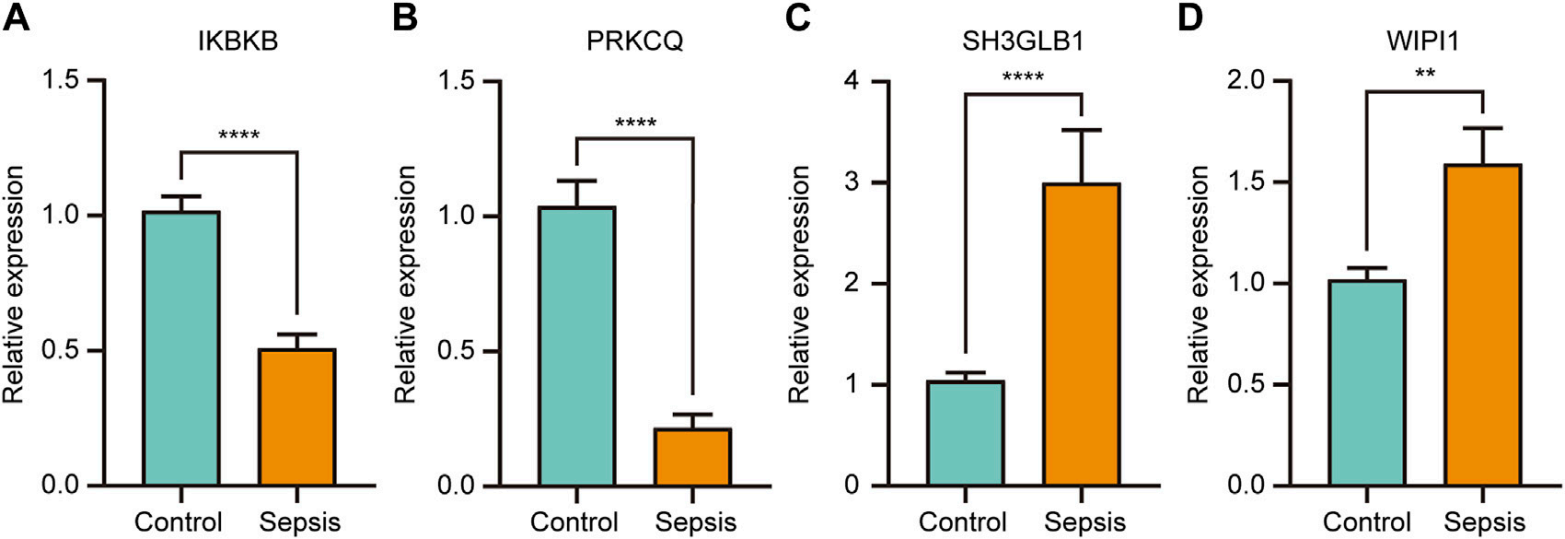
The relative expression of key genes with both diagnostic and prognostic value was compared between healthy controls and sepsis patients. Student’s t-test or Mann-Whitney *U*-test were used to compare the differences between the two groups. **p < 0.01, ****p < 0.0001.

The four key genes that were identified to have both significant diagnostic and prognostic value in sepsis (IKBKB, PRKCQ, WIPI1, and SH3GLB1) were validated by RT-qPCR in whole blood samples obtained from sepsis patients and healthy controls. The results showed that IKBKB and PRKCQ were significantly downregulated in the sepsis group, while SH3GLB1 and WIPI1 were significantly upregulated (Figures 9A–D). The expression trend of these four genes between the two groups was consistent with the results of microarray analysis in GSE65682 and GSE134347 (Figures 6A, B).

## Discussion

Sepsis is the most common cause of death in intensive care patients, and its pathogenesis has not been fully understood. Increasing evidence suggests that autophagy plays an important role in sepsis. To further explore the pathogenesis of sepsis and search for biomarkers with diagnostic and prognostic value, we performed a comprehensive bioinformatics analysis of two sepsis related datasets. Through differential expression analysis and WGCNA, we obtained 14 autophagy-related key genes. Functional enrichment analysis further confirmed that these genes are related to autophagy. We then constructed TF-gene and ceRNA regulatory networks of key genes to clarify their molecular regulation mechanisms.

ARGs have been shown to be of great value in the diagnosis and/or prognostic evaluation of diseases such as cancer, dilated cardiomyopathy, acute myocardial infarction, diabetic nephropathy, pulmonary hypertension, dermatomyositis, and rheumatoid arthritis (Du et al., 2020; Tong et al., 2021; Bai et al., 2022; Fan et al., 2022; Wang et al., 2022; Yang et al., 2022; Zhang et al., 2022). Therefore, ROC curve and survival analysis were performed on key genes to explore their diagnostic efficacy and prognostic value in sepsis. After analysis, we obtained 11 key genes with diagnostic efficacy and 5 key genes with prognostic value. Among them, IKBKB, PRKCQ, SH3GLB1 and WIPI1 were key genes with both diagnostic efficacy and prognostic value.

IKBKB (IKKβ) is a serine kinase that forms the IκB kinase (IKK) complex together with IKKα and IKKγ. IKK complex phosphorylates IκBs, an inhibitor of NF-κB, causing dissociation of the inhibitor and activation of NF-κB. Under different environmental conditions, activated NF-κB exerts either promoting or inhibiting effects on autophagy (Djavaheri-Mergny et al., 2006; Nivon et al., 2009; Jiang et al., 2011). In addition, the IKK complex has also been found to promote autophagy in an NF-κB independent manner under the stimulation of physiological and pharmacological factors (Criollo et al., 2010). On the other hand, IKKβ activity can also be downregulated by autophagic degradation, suggesting a complex interplay between autophagy and the IKK/NF-κB pathway (Qing et al., 2006; Niida et al., 2010). Ellipticine, an IKKβ inhibitor, was found to have anti-inflammatory properties by promoting autophagy in LPS-treated bone marrow-derived macrophages (Chen et al., 2019).

PRKCQ (PKCθ) is one of the PKC family members that is involved in the activation of AP-1 and NF-κB. It positively regulates autophagy in mouse skeletal muscle cells under endoplasmic reticulum (ER) stress and rat hepatic stellate cells under hypoxia (Madaro et al., 2013; Jin et al., 2016). In infection and sepsis, relevant studies have suggested that PKCθ mainly plays a pro-inflammatory role, but its effect on autophagy remains unclear. In mice infected with *salmonella*, PKCθ has been proved to promote a potent pro-inflammatory phenotype of macrophages to exert protective antimicrobial immunity (Pfeifhofer-Obermair et al., 2016). Besides, inhibition of PKCθ can inhibit Th17 cell response through the Notch signaling pathway, thereby alleviating acute lung injury in mice (Li et al., 2019).

SH3GLB1 (Bif-1) has multiple functions and is involved in autophagy, apoptosis and mitochondrial function. Under starvation conditions, Bif-1 has been demonstrated to promote the activation of PI3KC3 by forming a complex with Beclin1 through UVRAG. Then PI3KC3 regulates the formation of Atg9 puncta by mediating Golgi membrane fission to achieve the biogenesis of autophagosomes (Takahashi et al., 2007; Takahashi et al., 2011). According to a recent study, SH3GLB1 has diagnostic significance for pediatric sepsis (Zhang et al., 2021).

WIPI1 is a member of the human WIPI family and is similar to yeast Atg18. WIPI1, together with WIPI2, acts as a key effector of PtdIns3P in the nascent autophagosome to bridge PtdIns3P production and LC3 lipidation (Proikas-Cezanne et al., 2015; Almannai et al., 2022). Based on the positive regulation of autophagy by WIPI1, quantifying the expression of WIPI1 mRNA or the number of WIPI1 puncta is considered a reliable method to assess the level of autophagosome formation (Proikas-Cezanne et al., 2007; Tsuyuki et al., 2014). Consistent with our findings, in another bioinformatics study on ferroptosis-related genes, WIPI1 was found to be elevated in sepsis patients and correlated with patient outcomes (Zhu et al., 2022).

By analyzing the signatures of immune cell, we identified 11 significantly different immune cell subtypes in sepsis. These potential differences in immune cell abundance are consistent with many previous studies. Neutrophils and monocyte-macrophages are often found to be elevated in sepsis. This phenomenon is thought to be mainly associated with inhibition of apoptosis in neutrophils and monocyte-macrophages. The release of immature neutrophils is also thought to be involved. Although the numbers of these two immune cell types do not decrease, there is significant alteration in cellular function (Vaki et al., 2011; Delano and Ward, 2016). In addition, mast cells were found to be locally and systematically activated in a CLP-induced mouse model of septic peritonitis and TLR4 was shown to mediate LPS-induced mast cell activation (Supajatura et al., 2001; Seeley et al., 2011). On the contrary, sepsis leads to the reduction in CD4^+^ T cells, γδ T cells, CD8^+^ T cells, B cells, NK cells and dendritic cells by accelerating apoptosis. Of course, this reduction in numbers is accompanied by a decline in cell function (Rimmelé et al., 2016; Cao et al., 2019). In our study, except for γδ T cells, the changes of other immune cells were consistent with the findings described above. γδ T cells are critical responders and cytokine producers in the early stages of infection (Ferrick et al., 1995). During this period, γδ T cells increase dramatically in the blood and can be as high as 60% of total T cells (Chien et al., 2014). However, during later stages of sepsis, circulating γδ T cells are reduced by up to 80% (Kim and Oldham, 2019). Therefore, we speculate that the increased γδ T cells in the sepsis group in this study may be related to the fact that the early expanded cells have not completely fallen below the normal level.

Further correlation analysis showed that CD8^+^ T cell, resting memory CD4^+^ T cell, M1 macrophage and naive B cell signatures were associated with most key genes. Thus, we speculate that the abundance of these four types of immune cells in sepsis may be regulated by autophagy. T cell autophagy has been demonstrated to reduce apoptosis and improve immunosuppression in sepsis (Oami et al., 2017). Enhanced autophagy facilitates inhibition of M1-like macrophage polarization and alleviation of inflammation (Xu et al., 2020). In addition, autophagy has been shown to be involved in the early stages of B cells development (Clarke and Simon, 2019), and sepsis has been proved to induce peripheral naive B cells reduction (Suzuki et al., 2016), but whether autophagy is involved in this reduction remains unclear.

The expression of autophagy-related key genes in circulating cells was significantly altered in patients with sepsis, some of which were associated with the diagnosis, prognosis and immune cell signatures in sepsis. Therefore, key genes and autophagy process may participate in the pathophysiological process of sepsis by regulating circulating immune cells.

Similar to our conclusions, two other papers have also confirmed the importance of ARGs in sepsis. Di et al. (2023) obtained hub ARGs with diagnostic value in sepsis through WGCNA, Cytoscape and ROC analysis, and identified their correlation with differentially infiltrated immune cells. Chen et al. (2022) used machine learning algorithms to identify hub ARGs based on survival outcomes and constructed an ARG classifier for early diagnosis, prognosis, and predicting immune microenvironment features in sepsis. Compared with the above studies, our advantages are reflected in the survival analysis based on survival time, the ceRNA network constructed based on sequencing results, and the TF-gene regulatory network based on prediction. And through our study, the importance of ARGs in sepsis has been further confirmed.

Although our study comprehensively analyzed the potential roles of peripheral blood ARGs in diagnosis, prognosis and immune cell signatures in sepsis. However, there are still some unavoidable limitations. Firstly, the datasets we analyzed were downloaded from the GEO, so detailed clinical data was not available. Secondly, due to the small number of clinical samples we collected, the diagnostic efficiency and prognostic value of the key genes have not been validated. Thirdly, because of the wide array of infectious agents and the diversity of severity and stage of sepsis, the key genes identified may not be representative of all forms of sepsis. Finally, Limited by the current data, the diagnostic and prognostic value of key genes in sepsis has not been compared with other diseases to clarify their specificity. Therefore, collecting more clinical specimens, constructing our own dataset and conducting more in-depth and comprehensive analysis will become one of our future research directions.

## Conclusion

This study shed light on the potential roles of peripheral blood ARGs in sepsis. Firstly, we identified 11 key genes with diagnostic efficiency and 5 key genes with prognostic value. Subsequently, we obtained and verified 4 genes including IKBKB, PRKCQ, WIPI1 and SH3GLB1, which had both diagnostic and prognostic value. Finally, the abundance of CD8^+^ T cells, resting memory CD4^+^ T cells, M1 macrophages and naive B cells were found to correlate with the expression levels of most key genes. These findings may facilitate the development of promising biomarkers for sepsis. More importantly, it reveals the important role of autophagy in the pathogenesis of sepsis and provides a strong impetus for more in-depth study.

## Data Availability

The original contributions presented in the study are included in the article/Supplementary Material, further inquiries can be directed to the corresponding author.
