# Supplementary material for: Diagnostic and prognostic value of autophagy-related key genes in sepsis and potential correlation with immune cell signatures
Source: Front Cell Dev Biol. 2023 Aug 28;11:1218379. doi: 10.3389/fcell.2023.1218379 (PMC10493283; doi:10.3389/fcell.2023.1218379)
Supplement: Supplementary file 1 [file Table1.DOCX]

**Supplementary Table 1** Characteristics of the individuals included in the validation experiment

| Variable | Control (n=11) | Sepsis (n=11) |
| --- | --- | --- |
| Age, years | 53.55 ± 14.74 | 59.36 ± 15.78 |
| Female/Male | 4 / 7 | 2 / 9 |
| APACHE II score | NA | 19.82 ± 7.08 |
| SOFA score | NA | 9.36 ± 3.32 |
| Lactate, mmol/L | NA | 2.20 (1.40-3.80) |
| PCT, ng/ml | NA | 7.89 (2.93-18.61) |
| WBC, 10^9^/L | NA | 16.30 ± 4.47 |
| Site of infection, n (%) |  |  |
| Lung | NA | 3 (27.27) |
| Abdomen | NA | 4 (36.36) |
| Urinary tract | NA | 1 (9.09) |
| Blood | NA | 1 (9.09) |
| Other | NA | 2 (18.18) |
| Comorbidities, n (%) |  |  |
| Hypertension | NA | 1 (9.09) |
| Heart disease | NA | 1 (9.09) |
| Diabetes | NA | 2 (18.18) |
| Cancer | NA | 5 (45.45) |
| Nervous system disease | NA | 2 (18.18) |
| Autoimmune disease | NA | 3 (27.27) |

Normally distributed variables are presented as the mean ± standard deviation, and non-normally distributed variables are presented as the median (quartiles).

### APACHE, Acute Physiology and Chronic Health Evaluation; SOFA, Sequential Organ Failure Assessment; PCT, proealcitonin; WBC, white blood cell.
