# Supplementary material for: Diagnostic and prognostic value of autophagy-related key genes in sepsis and potential correlation with immune cell signatures
Source: Front Cell Dev Biol. 2023 Aug 28;11:1218379. doi: 10.3389/fcell.2023.1218379 (PMC10493283; doi:10.3389/fcell.2023.1218379)
Supplement: Supplementary file 2 [file Table2.docx]

**Supplementary Table 2** The primer sequences used for RT-qPCR.

| Gene | Forward primer (5’→3’) | Reverse primer (5’→3’) |
| --- | --- | --- |
| IKBKB | ACAGCGAGCAAACCGAGTTTGG | CCTCTGTAAGTCCACAATGTCGG |
| PRKCQ | GCATCCGTTTCTGACGCACATG | CGCTCTGGAAAGGTCGAACTTG |
| WIPI1 | CTTCAAGCTGGAACAGGTCACC | CGGAGAAGTTCAAGCGTGCAGT |
| SH3GLB1 | CCCATCACCTTCGCTGTCTGAA | GGTACAGGTGTCACAGAAGTCTG |
| ACTB | CACCATTGGCAATGAGCGGTTC | AGGTCTTTGCGGATGTCCACGT |
