# Supplementary material for: Diagnostic and prognostic value of autophagy-related key genes in sepsis and potential correlation with immune cell signatures
Source: Front Cell Dev Biol. 2023 Aug 28;11:1218379. doi: 10.3389/fcell.2023.1218379 (PMC10493283; doi:10.3389/fcell.2023.1218379)
Supplement: Supplementary file 3 [file Table3.DOCX]

**Supplementary Table 3** Results of GO analysis.

| ID | Description | GeneRatio | BgRatio | *p*.adjust | Count |
| --- | --- | --- | --- | --- | --- |
| BP:0009267 | cellular response to starvation | 5/14 | 160/20709 | 6.01E-05 | 5 |
| BP:0042594 | response to starvation | 5/14 | 196/20709 | 8.28 E-05 | 5 |
| BP:0031669 | cellular response to nutrient levels | 5/14 | 213/20709 | 8.35 E-05 | 5 |
| BP:0031668 | cellular response to extracellular stimulu | 5/14 | 245/20709 | 0.000125 | 5 |
| BP:0071496 | cellular response to external stimulus | 5/14 | 309/20709 | 0.000316 | 5 |
| BP:0018209 | peptidyl-serine modification | 5/14 | 338/20709 | 0.000409 | 5 |
| BP:0042149 | cellular response to glucose starvation | 3/14 | 50/20709 | 0.000831 | 3 |
| BP:0031667 | response to nutrient levels | 5/14 | 446/20709 | 0.001187 | 5 |
| BP:0009991 | response to extracellular stimulus | 5/14 | 479/20709 | 0.001492 | 5 |
| BP:0007183 | SMAD protein complex assembly | 2/14 | 13/20709 | 0.004051 | 2 |
| CC:0005776 | autophagosome | 3/14 | 112/20709 | 0.005074 | 3 |
| CC:0016235 | aggresome | 2/14 | 35/20709 | 0.011785 | 2 |
| CC:0000421 | autophagosome membrane | 2/14 | 51/20709 | 0.017172 | 2 |
| CC:0016234 | inclusion body | 2/14 | 74/20709 | 0.024912 | 2 |
| CC:0005774 | vacuolar membrane | 3/14 | 449/20709 | 0.039412 | 3 |
| CC:1904813 | ficolin-1-rich granule lumen | 2/14 | 124/20709 | 0.039412 | 2 |
| MF:0045296 | cadherin binding | 4/14 | 333/20709 | 0.005080 | 4 |
| MF:0030331 | nuclear estrogen receptor binding | 2/14 | 39/20709 | 0.013607 | 2 |
| MF:0046332 | SMAD binding | 2/14 | 78/20709 | 0.025429 | 2 |
| MF:0106310 | protein serine kinase activity | 3/14 | 360/20709 | 0.028072 | 3 |
| MF:0004674 | protein serine/threonine kinase activity | 3/14 | 430/20709 | 0.039316 | 3 |
| MF:0004712 | Protein serine/threonine/tyrosine kinase activity | 3/14 | 446/20709 | 0.039412 | 3 |
| MF:0031072 | heat shock protein binding | 2/14 | 123/20709 | 0.039412 | 2 |
| MF:0140297 | DNA-binding transcription factor binding | 3/14 | 470/20709 | 0.041868 | 3 |
| MF:0002020 | protease binding | 2/14 | 136/20709 | 0.042492 | 2 |
| MF:0016922 | nuclear receptor binding | 2/14 | 139/20709 | 0.042936 | 2 |
