# Supplementary material for: Diagnostic and prognostic value of autophagy-related key genes in sepsis and potential correlation with immune cell signatures
Source: Front Cell Dev Biol. 2023 Aug 28;11:1218379. doi: 10.3389/fcell.2023.1218379 (PMC10493283; doi:10.3389/fcell.2023.1218379)
Supplement: Supplementary file 4 [file Table4.docx]

**Supplementary Table 4** Results of KEGG analysis.

| ID | Description | GeneRatio | BgRatio | *p*.adjust | Count |
| --- | --- | --- | --- | --- | --- |
| hsa05131 | Shigellosis | 6/13 | 247/8190 | 5.52E-05 | 6 |
| hsa04210 | Apoptosis | 5/13 | 136/8190 | 5.52E-05 | 5 |
| hsa04140 | Autophagy - animal | 5/13 | 141/8190 | 5.52E-05 | 5 |
| hsa04064 | NF-kappa B signaling pathway | 4/13 | 104/8190 | 4.10E-04 | 4 |
| hsa05162 | Measles | 4/13 | 139/8190 | 0.001029 | 4 |
| hsa04141 | Protein processing in endoplasmic reticulum | 4/13 | 171/8190 | 0.001925 | 4 |
| hsa05417 | Lipid and atherosclerosis | 4/13 | 215/8190 | 0.003994 | 4 |
| hsa05145 | Toxoplasmosis | 3/13 | 112/8190 | 0.008216 | 3 |
| hsa01523 | Antifolate resistance | 2/13 | 30/8190 | 0.011183 | 2 |
| hsa04217 | Necroptosis | 3/13 | 159/8190 | 0.018154 | 3 |
| hsa05010 | Alzheimer disease | 4/13 | 384/8190 | 0.022502 | 4 |
| hsa04621 | NOD-like receptor signaling pathway | 3/13 | 184/8190 | 0.022970 | 3 |
| hsa05134 | Legionellosis | 2/13 | 57/8190 | 0.027727 | 2 |
| hsa04920 | Adipocytokine signaling pathway | 2/13 | 69/8190 | 0.035961 | 2 |
| hsa05022 | Pathways of neurodegeneration - multiple diseases | 4/13 | 476/8190 | 0.035961 | 4 |
| hsa05132 | Salmonella infection | 3/13 | 249/8190 | 0.040380 | 3 |
| hsa05235 | PD-L1 expression and PD-1 checkpoint pathway in cancer | 2/13 | 89/8190 | 0.048224 | 2 |
| hsa04658 | Th1 and Th2 cell differentiation | 2/13 | 92/8190 | 0.048224 | 2 |
| hsa05222 | Small cell lung cancer | 2/13 | 92/8190 | 0.048224 | 2 |
